# Targeted vs. full population screening costs for incident atrial fibrillation and AF-related stroke for a healthy population aged 65 years in the United Kingdom

**DOI:** 10.1093/ehjqcco/qcac005

**Published:** 2022-02-09

**Authors:** Paul Burdett, Gregory Y H Lip

**Affiliations:** Liverpool Centre for Cardiovascular Science, University of Liverpool, L7 8TX, United Kingdom; Liverpool Centre for Cardiovascular Science, University of Liverpool, L7 8TX, United Kingdom

**Keywords:** Atrial Fibrillation, Forecast, Screening costs, National Health Service (NHS)

## Abstract

**Aims:**

Atrial Fibrillation (AF) is the most common sustained heart arrhythmia and a major preventable cause of stroke. Stroke accounts for a large amount of health and social care funding and over the coming years is likely to place an increasing cost burden on the wider UK health care system. We therefore need to understand how an opportunistic AF screening programme would impact on healthcare costs of AF (and AF-related stroke) for the NHS.

**Methods and results:**

Using UK population forecasts and prior published data we initially calculated the number of people to be screened, newly-diagnosed and treated for Atrial Fibrillation (AF), and the associated costs of such a programme for all 65 year olds and for just a ‘high risk’ group. The reduction in the number of stroke cases recorded and the associated cost savings were subsequently calculated, for 2020 and the projected estimates over the following decade. The number of newly diagnosed AF patients at 65 years old for the two groups (all 65 year olds and for just a ‘high risk’ group) would be in 6754 and 797 in 2020, rising to 9200 and 1086 in 2030, respectively. In 2020 the cost of the screening programme for the two options would be £14.3m and £1.7m. If AF is medicated and monitored then there would be a subsequent reduction in the number of stroke cases in 2020 by 4323 or 510 depending on the group screened, with associated savings of £394.2m and £46.5m,  respectively. Focussing on 2030 and should opportunistic screenings for AF be introduced at age 65, with subsequent treatment, it is predicted to reduce the number of stroke cases over the decade by 5888 if all 65 year olds are screened and 695 if just the high risk group are screened. If the number of strokes can be reduced by treating these screened AF patients, we would substantially reduce the health and social care costs of stroke by £654.6m and £77.3m,  respectively.

**Conclusion:**

The number of newly diagnosed AF patients at age 65 will rise over the decade between 2020 and 2030. Screening and treatment of AF will substantially reduce the health and social care costs of AF-related stroke in the NHS.

**What is already known about this subject?** Costs of AF and associated cases of stroke are already known to be a large burden on NHS and social care budgets, and previous studies have provided some estimates.

**What does this study add?** This study seeks to understand more about whether a targeted ‘high risk’ group or full population screening programme at age 65 would be the preferred method of detecting previously undiagnosed AF. We are looking to analyse and forecast the screening costs, and possible savings through prevention, of AF and stroke over the coming years.

**How might this impact on clinical practice?** Such data and forecast information would help with NHS resource and social care planning over the next decades.

## Introduction

Atrial fibrillation (AF) is an irregular and often very rapid heart rhythm (arrhythmia). It is the most common sustained heart arrhythmia and a major preventable cause of stroke, heart failure, and dementia.  AF and stroke already account for a significant amount of National Health Service (NHS) funding, and over the coming years is highly likely to impose a growing cost on NHS budgets and the wider UK health care system.^1,2^

AF is often asymptomatic, and it is estimated that only 1 in 12 episodes of paroxysmal AF are symptomatic, in contrast to other supraventricular tachycardia.^3^ Nevertheless, asymptomatic patients are at similar or even higher risk of adverse outcomes, in comparison with symptomatic AF.^4^ Thus, screening for AF has been proposed by many experts,^5,6^ although authorities have not formally recommended routine AF screening as such a strategy is yet to be proven to positively impact clinical outcomes.

Approximately 1 in 100 of the population have AF, though the prevalence increases to more than 1 in 10 in elderly people.^7^ The prevalence and incidence rate of AF are increasing; due to an aging population and in increasing survival rates from conditions associated with AF, such as hypertension and heart failure.^8,9^ Nonetheless, the prognosis especially amongst the elderly has not been improving over the last decade.^8^

There is some debate whether AF screening of all 65 year olds should be conducted, compared to more targeted screening of ‘high risk’ individuals (for example, as determined by the C2HEST Risk Score) in a healthy population (at age 65). The Screening for AF in the Elderly (SAFE) study suggested that systematic and opportunistic screening were similarly good at identifying prevalent AF in the population, but opportunistic screening was more cost effective.^10^ An analysis from the Huawei Heart Study suggested that more targeted screening with the C2HEST Risk Score, especially in association with symptoms, improved the detection of AF in a general population screening study using mobile health (mHealth) technology.^11^ Indeed, the latter has expanded, with the availability of smartwear and novel mHealth solutions to detect AF.^12^

Stroke accounts for a large amount of health and social care funding and over the coming years is likely to place an increasing cost burden on the wider UK health care system. The aim of this study is to investigate how an opportunistic AF screening programme would impact on healthcare costs of AF (and AF-related stroke) for the NHS.

## Methods

This cost modelling exercise would assume a community based screening program with initial screenings being conducted at GP surgeries, with patients being identified with possible AF being referred onto specialist clinics for further investigation.

We first determined the forecasted total population of all 65 year olds that would be screened if total population were screened and the total if just ‘high risk’ patients, as determined by the C2HEST Risk Score, were to be targeted. Using the forecasted populations for 2020–2030 based on ONS projections^13^ we were able to estimate the expected total population of 65 year olds for each year of the study.

Based on a prior study from Denmark,^14^ which was a nationwide cohort study of all Danish citizen aged ≥65 years that evaluated the performance of the C2HEST score, we were able to determine the ‘high risk’ population of 65 year olds that could be targeted for screening. ‘High risk’ subjects were defined as having a 5 year risk of new onset AF of 11.8% at age 65; and we have classed this group as our ‘high risk’ targeted screening group. It should be acknowledged that any modelling of this type makes assumptions, and using data from Denmark^15^ may marginally inflate the overall cost of AF in the UK.

In order to calculate the total healthy populations to be screened we excluded already known cases of AF. We estimated this to be 2% of 65 year olds based on a prior study, the ‘SAFE’ study,^10^ which determined the most cost-effective method of screening for AF in the population aged 65 years and over, as well as its prevalence and incidence in this age group.

Screening costs were based prior published data of what equipment and resources would be required for each patient screened, they include Kardia,^16^ pulse palpation readings,^10^ and 12-lead ECGs.^17^ Based on Berge et al.^18^ and Quality and Outcomes Framework AF Prevalence National Cardiovascular Intelligence Network (NCVIN) Public Health England (PHE) data^19^ we estimated that the proportion of people that will have a new AF diagnosis will be approximately 1% for both groups. Using these AF prevalence forecasts and combined with screening costs we then calculated the expenditure required to screen both population groups from 2020–2030.

Based on a prior study,^2^ medication and monitoring costs—that include annual prescriptions for Anticoagulants and visits to clinic—were calculated using a prevalence based approach to estimate the cost associated to each newly diagnosed AF patient. It is assumed a rate of seven prescriptions per patient per year with each of those patients requiring eight annual visits to an anticoagulant clinic, for those patients on Warfarin. It is likely that the use of Warfarin will reduce over time, along with associated anticoagulation clinic visits. Conversely, the likely usage of drugs in the NOACs (Non-VKA Oral Anticoagulants) class, will have an associated rise in patient numbers linked to the similar reduction in Warfarin usage over the coming years. There is also likely to be an increase in the number of OPD monitoring visits, for example, for renal function testing.

Detection of AF and subsequent treatment and care, particularly with oral anticoagulants leads to an approximately 64% reduction in the likelihood of a stroke event^20^ (the main AF-related complication focused in our analysis) occurring in that patient, compared to leaving such patients untreated.

Estimates for the annual cost of each stroke case have been based upon figures produced by the Stroke Association^17^ and represent the total societal cost of AF-related stroke in the first year and in subsequent years, conditional on survival.

## Patient and public involvement

This is a financial cost analysis and forecast of AF and Stroke prevention, and as such it has not been relevant to involve patients in this study.

## Results

In 2020 the modelling shows that the screenings could lead to newly diagnosed cases of AF, for both groups, that is all 65 year olds and just ‘high risk’ 65 year olds, of 6754 and 797 people, respectively.

**Figure 1:**
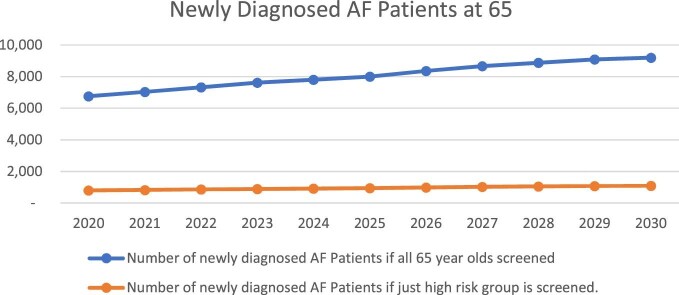
Newly diagnosed atrial fibrillation patients.

In 2025 and 2030 new cases for both groups would be 7999 and 944, and 9200 and 1086, respectively. *Figure 1* highlights the growing numbers of new AF cases for both groups of age 65 year olds to be screened.

*Table 1* *A* shows that in 2020 the estimated cost for AF screening of both groups, the total screening costs would be £14.3m and £1.7m,  respectively. For 2025 and 2030, the modelling would mean that the costs of screening for both groups would be £18.7m and £2.2m,  and £23.8m and £2.8m,  respectively.

**Table 1:** Screening, costs, and impact on atrial fibrillation related stroke

**(a) Number of newly diagnosed AF patients**			
**Newly diagnosed AF patients**	2020	2025	2030
Number of newly diagnosed AF Patients if all 65 year olds screened	6754	7999	9200
Number of newly diagnosed AF Patients if just high risk group is screened	797	944	1086
**(b) Total costs of screening**			
	2020	2025	2030
(i) Kardia–unit cost per patient	0.32	0.35	0.39
(ii) Pulse reading	3	3.31	3.66
(iii) 12-lead ECG interpreted by a consultant	16.91	18.67	20.61
(iv) Other costs of screening programme	1	1.10	1.22
Total cost per patient (£)	**21.22**	**23.43**	**25.87**
Total screening cost for aLL 65 year olds less already known AF cases (£)	**14 334 110**	**18 742 562**	**23 799 774**
Total screening cost for HIGH RISK 65 year olds less already known AF cases(£)	**1 691 425**	**2 211 622**	**2 808 373**
Cost difference between screening costs ALL vs. High Risk only (£)	**12 642 685**	**16 530 939**	**20 991 400**
**(c) Annual and cumulative medication & monitoring costs for newly diagnosed AF patients**			
	**2020**	**2025**	**2030**
Total Cost medication & monitoring for all 65 year olds (£)	**1 351 909**	**1 767 688**	**2 244 654**
Total cost medication & monitoring for HIGH RISK 65 year olds only (£)	**159 525**	**208 587**	**264 869**
Cost difference between Medication & monitoring costs all vs. High Risk only (£)	**1 192 384**	**1 559 101**	**1 979 785**
**Cumulative M&M costs**			
For All 65 year olds	**1 351 909**	**9 391 562**	**19 772 665**
For High RISK 65 year olds only	**159 525**	**1 108 204**	**2 333 174**
**If AF is medicated, there is a reduction of Stroke by 64%**	2020	2025	2030
**(d) Numbers of stroke cases prevented due to screenings and cost savings**			
64% reduction in stroke cases if all 65 year olds screened	4323	5119	5888
64% reduction in stroke cases if High Risk 65 year olds screened only	510	604	695
Total cost of stroke (£)	91 212	100 705	111 187
cost savings due to stroke prevention if All 65 year olds screened (£)	394 277 539	515 537 506	654 642 421
Cost savings due to stroke prevention if HIGH RISK 65 year olds screened only (£)	46 524 750	60 833 426	77 247 806

**Figure 2:**
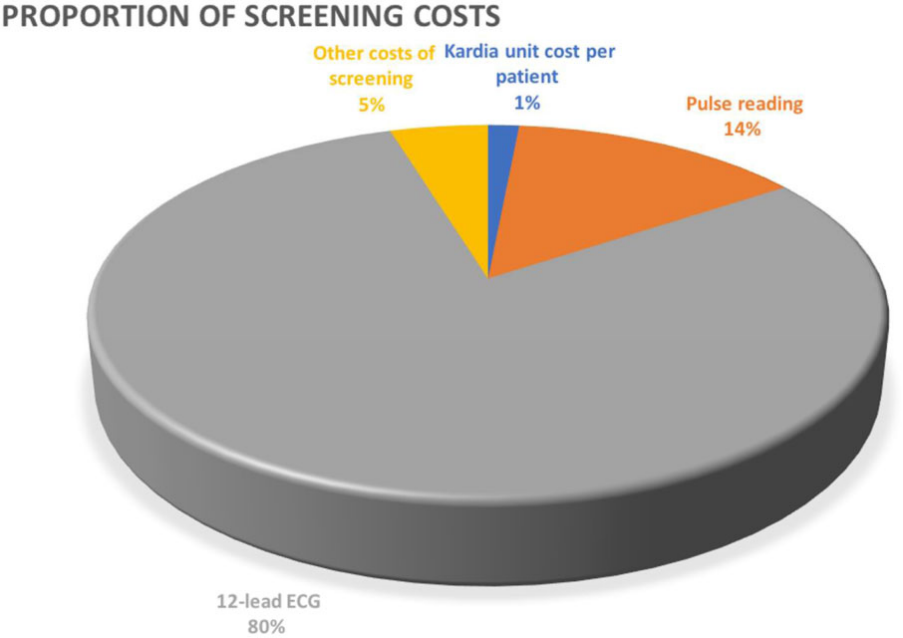
Screening cost category split.

*Figure 2* and *Table 1**B* both illustrate the costs involved for the screening programme. *Table 1**B* contains the total screening costs for both groups, while *Figure 2* highlights the proportional split of screening costs across different cost categories. This includes use of Kardia unit cost per patient (in 2020£0.32 and in 2030£0.39), pulse readings (£3 and £3.66 in 2020 and 2030, respectively), 12 lead ECG interpreted by a Consultant (£16.91 in 2020, rising to £20.61 in 203), and other costs such as promotion and administration of the screening programme (estimated at £1 in 2020, and £1.22 in 2030). It highlights that the 12 lead ECG readings are the major driver of costs and as such could be used as an aid to future planning and resource management.

**Figure 3:**
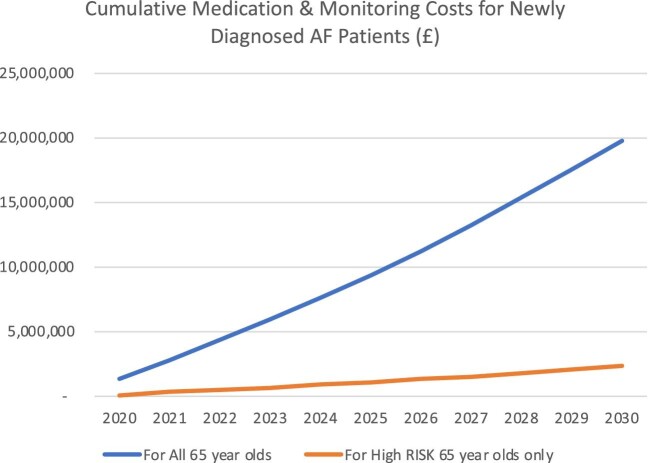
Cumulative medication & monitoring costs for newly diagnosed atrial fibrillation patients.

*Table 1* *C* and *Figure 3* both illustrate the growing annual cost of AF medication and monitoring due to an increasingly growing elderly population, and also the increasing cumulative annual costs that would be needed for the newly diagnosed AF patients due to the proposed screening programmes.

In 2020 the annual cost for the two groups would be £1.35m and £0.16m,  increasing to £2.24m and £0.26m by 2030, respectively. Cumulatively these costs for the two groups would be £19.77m and £2.33m by 2030, respectively.

**Figure 4:**
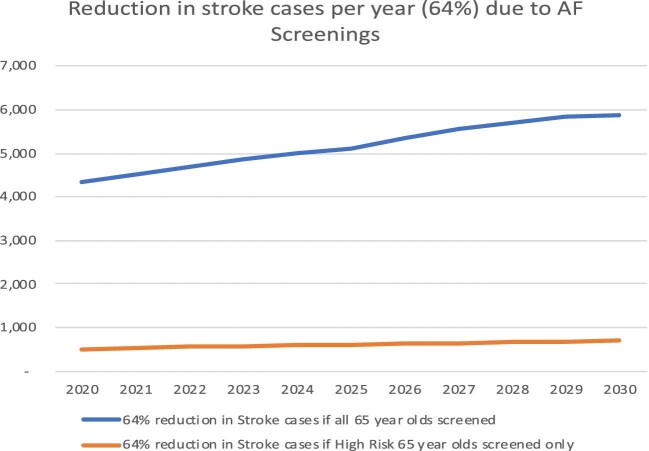
Reduction in strokes due to atrial fibrillation screening programmes.

*Table 1* *D* and *Figure 4* shows the number of stroke cases that would be prevented from occurring in both screening groups. In 2020 the number of prevented strokes would be 4323 and 510, and in 2030 would increase to 5888 and 695 for the two groups, respectively.

Based on previous analysis provided by the Stroke Association, we have estimated the mean cost of stroke with NIHSS score 3 (moderate stroke) to be £59 181, which includes informal care and lost employment in year 1 from the perspective of NHS and personal social services (PSS) in 2020. In subsequent years post-stroke, patients with AF are estimated to result in £32 031, in NHS and PSS costs, informal care, and lost productivity (at 2020 costs). The total societal cost of AF-related stroke is projected to be £91 212 conditional on survival (at 2020 levels).

**Figure 5:**
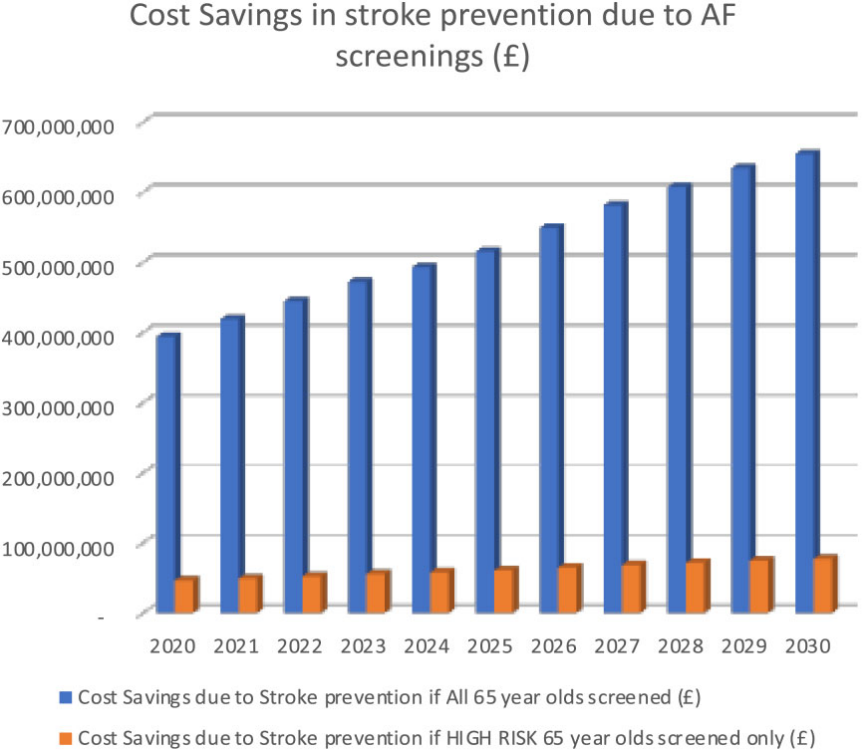
Cost savings due to atrial fibrillation screenings.

*Table 1* *D* and *Figure 5* both highlight the increasing societal cost savings due to prevented strokes each year that could be achieved whichever of the two screening programmes is introduced. In 2020, £394.28m and £46.52m could be saved from the two groups by detecting AF and initiating treatment (i.e. stroke prevention), increasing in 2030 to £654.64m and £77.25m,  respectively.

## Discussion

This analysis highlights the cost burden to the NHS of AF and AF-related strokes, emphasizing the current and projected future healthcare costs, both direct and indirect, related to strokes linked to AF.

Undiagnosed and asymptomatic AF carries a similar or even worse prognosis compared to symptomatic AF, and often the first presentation of AF may be with an associated complication, such as stroke or heart failure.

Focussing on 2020, we found that the annual cost of strokes could be reduced by over £394m or £46m (depending on the group screened) with the correct diagnosis, medication, and monitoring. These costs are forecast to increase over the subsequent years and decades.

If AF can be detected earlier and the number of strokes can be reduced, we would substantially reduce the healthcare costs of both conditions to the NHS and wider society. Improved screening strategies to improve this detection and subsequent stroke events are urgently needed. The application of a more complete approach to AF care has been associated with reduced health care costs. This is important given that AF management is more than simply stroke prevention, as reflected in recent clinical guidelines promoting a more holistic or integrated approach to AF care,^21^ based on the ABC (Atrial fibrillation Better Care) pathway.^22^ Indeed, adherence to the ABC pathway is associated not only with a reduction in stroke risk, but with a major impact in reducing all cause mortality, cardiovascular mortality, major bleeding, and hospitalizations.^23^ Indeed, the importance of an integrated care approach to chronic long term conditions is increasingly recognized.^24,25^

Hospitalizations are a major driver of AF-related conditions, especially when associated with other multimorbidity issues.^2^ Many patients present with AF for the first time, in the context of decompensated heart failure or stroke. Such associated comorbid conditions related to AF remain highly prevalent and increasing as the UK population profile ages and multimorbidity increases with incident risk factors.^26–29^

It should also be recognized that while screening for AF is yet to be approved in countries such as the USA,^30^ we believe that it is important and useful to be able to demonstrate the potential impact a screening programme could have on a population and healthcare system.

## Limitations

Similar to other studies, it should be acknowledged that this analysis too has its limitations. Assumptions have been based on the profile of AF patients and costs remaining reasonably constant over the period studied. Due to an ageing population the prevalence of AF is likely to *increase* year-on-year. Annual forecasts for the rate of inflation are based on estimates, which in reality will be subject to change. The cost of medications such as NOACs may reduce over the period, however, there may be an increased use of these drugs amongst AF patients.

This is a complex, initial analysis of AF and associated stroke costs. In order to completely comprehend and gain insights as to whether opportunistic screening programmes would provide a good investment of resources would require further analysis and include more detailed modelling.

We have focussed on providing some simple modelling analyses assuming medication non-adherence of 20% in the first year with NOACs and the impact of costs.^31^ Also, the impact of medication intolerance being 10% per year. The data would also suggest that anticoagulation is beneficial in AF patients with frailty, at risk of falls and have a prior bleed, as well as those with valve disease.^32–34^ Finally, our focus is AF screening and detection, whether in primary care or hospitals—not interventions.

Despite these limitations, this modelling illustrates the growing number of people in the population who would likely benefit from opportunistic screenings at 65. Early diagnosis and treatment of AF, will in turn have an impact on the individual health outcomes, the NHS, health & social provision, and the economy and society in the UK.

## Conclusion

The number of newly diagnosed AF patients at age 65 will rise over the decade between 2020 and 2030. Screening and treatment of AF will substantially reduce the health and social care costs of AF-related stroke in the NHS.

## Data availability

The data underlying this article are available in the article and in its online supplementary material.
